# Host responses influence on the induction of lambda prophage

**DOI:** 10.1111/j.1365-2958.2008.06119.x

**Published:** 2008-04-01

**Authors:** Assaf Rokney, Oren Kobiler, Amnon Amir, Donald L Court, Joel Stavans, Sankar Adhya, Amos B Oppenheim

**Affiliations:** 1 Department of Molecular Genetics and Biotechnology, The Hebrew University–Hadassah Medical School Jerusalem, Israel; 2 Department of Physics of Complex Systems, Weizmann Institute of Science Rehovot, Israel; 3 Gene Regulation and Chromosome Biology Laboratory, National Cancer Institute at Frederick Frederick, MD, USA; 4 Laboratory of Molecular Biology, National Cancer Institute Bethesda, MD, USA

## Abstract

Inactivation of bacteriophage lambda CI repressor leads almost exclusively to lytic development. Prophage induction can be initiated either by DNA damage or by heat treatment of a temperature-sensitive repressor. These two treatments also cause a concurrent activation of either the host SOS or heat-shock stress responses respectively. We studied the effects of these two methods of induction on the lytic pathway by monitoring the activation of different lambda promoters, and found that the lambda genetic network co-ordinates information from the host stress response networks. Our results show that the function of the CII transcriptional activator, which facilitates the lysogenic developmental pathway, is not observed following either method of induction. Mutations in the *cro* gene restore the CII function irrespective of the induction method. Deletion of the heat-shock protease gene *ftsH* can also restore CII function following heat induction but not following SOS induction. Our findings highlight the importance of the elimination of CII function during induction as a way to ensure an efficient lytic outcome. We also show that, despite the common inhibitory effect on CII function, there are significant differences in the heat- and SOS-induced pathways leading to the lytic cascade.

## Introduction

**Fig. 1:**
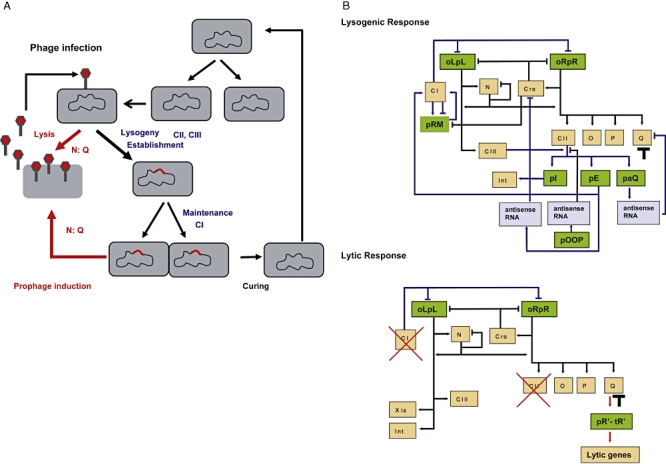
Lifestyles of phage λ. A. A cell infected with phage may follow the lytic or lysogenic response. The lysogenic state can be maintained indefinitely (prophage shown in red), occasionally cured and become non-lysogen or induced leading to lytic development. B. A schematic diagram showing the key regulatory elements in the lytic and lysogenic response. In both the lytic and lysogenic response, early transcription of N and Cro take place. In the lytic pathway, the delayed early transcripts lead to the accumulation of Q, which activates late gene expression (head and tail genes and lysis functions). CII activity is maintained below a detectable level (this work). In contrast, during the lysogenic response, CII activity dominates the network. High levels of CII, which are stabilized by CIII, lead to the activation of transcription from the pI, pE and paQ promoters. The inhibition of Q expression by the transcript initiated at the paQ promoter prevents late gene expression. Promoters are shown in green, phage functions in orange, antisense RNA in purple and lysogenic, and lytic pathways are shown in blue and red respectively (adapted from Oppenheim *et al*., 2005). The threshold effect on Q activity is shown as a black T.

Temperate bacteriophage lambda exists in a symbiotic relationship with its host and can undergo either lytic or lysogenic development (Campbell, 2003; Livny and Friedman, 2004; Ptashne, 2004; Dodd *et al*., 2005) (Fig. 1A). The highly stable lysogenic state, in which the prophage replicates passively with the host genome, is maintained by the expression of the CI repressor that by binding at the oL and oR operators, blocks the cascade of phage lytic gene expression. In the repressed ‘off’ state, the CI repressor dominates the control of phage gene expression, while in the induced ‘on’ state, the CI repressor is inactivated. Treatment with DNA-damaging agents can lead to activation of the host SOS response, a DNA repair system induced by DNA damage. This results in the inactivation of the CI repressor and thus in an irreversible switch into lytic development (Campbell, 2003; Ptashne, 2004). In both the infection and induction processes, the λCro protein partially represses the pL and pR promoters, and its activity is required for efficient lytic growth (Svenningsen *et al*., 2005).

Prophage induction initiates the inactivation of the CI repressor (Fig. 1B). DNA damage following either UV irradiation or treatment with mitomycin C elicits the SOS response network, activating the co-protease function of RecA that promotes the auto-cleavage of the host SOS LexA repressor as well as the phage λCI repressor (Little, 1984). Prophage induction by heat treatment is caused by direct denaturation of a mutant, temperature-sensitive repressor (Svenningsen *et al*., 2005). This heat induction method has been used for many years to study λ gene regulation and has proven effective for studying temporal gene expression. Each method of induction leads to the same lytic outcome, but elicits different host genetic networks, concurrently with the execution of the phage lytic cascade. We have raised the question whether there is any interaction between the phage network and the host stress networks beyond the initial stage of repressor inactivation by SOS or heat induction.

Lysogeny is preferred when phage infects starved cells, or when phage infects at multiplicities of infection of two or more (Kourilsky and Knapp, 1974; Kobiler *et al*., 2005). A small number of phage functions are specifically required for the establishment of a highly stable lysogenic state (Dodd *et al*., 2005; Oppenheim *et al*., 2005). A critical function is provided by the λCII protein. CII stimulates rapid synthesis of Int protein and of CI repressor from the pI and pE (also called pRE) promoters respectively. CII also activates the antisense paQ promoter, which reduces the Q anti-termination function and thus late gene expression (Fig. 1B; also summarized in Oppenheim *et al*., 2005). CII is a very unstable protein that is degraded primarily by the ATP-dependent protease FtsH (also known as HflB), which is found in complex with the HflKC proteins (Ito and Akiyama, 2005). Effectively, higher CII levels are achieved when the λCIII protein, an inhibitor of FtsH, is present. CIII, like CII, favours the establishment of lysogeny (Oppenheim *et al*., 2005), and is not required for lytic development. As expected, excess amounts of CII or CIII inhibit lytic development through activation of phage pI, pE and paQ promoters (Belfort and Wulff, 1973a; Belfort *et al*., 1975; Kornitzer *et al*., 1991). Based on these findings, one may predict that CII activity needs to be restricted for an effective lytic switch after induction.

In order to study gene regulation following induction, we monitored the activities of CI, CII and Q, using relevant λ promoters fused to the green fluorescent protein (GFP; Kobiler *et al*., 2005; Amir *et al*., 2007). Prophage induction was carried out by DNA-damaging agents or by heat treatment. We found that in spite of the same lytic outcome, the two induction processes lead to differences in the regulation of the phage pR, pE and pR′-tR′ promoters during lytic development.

## Results

### Prophage gene expression following heat induction

**Fig. 2:**
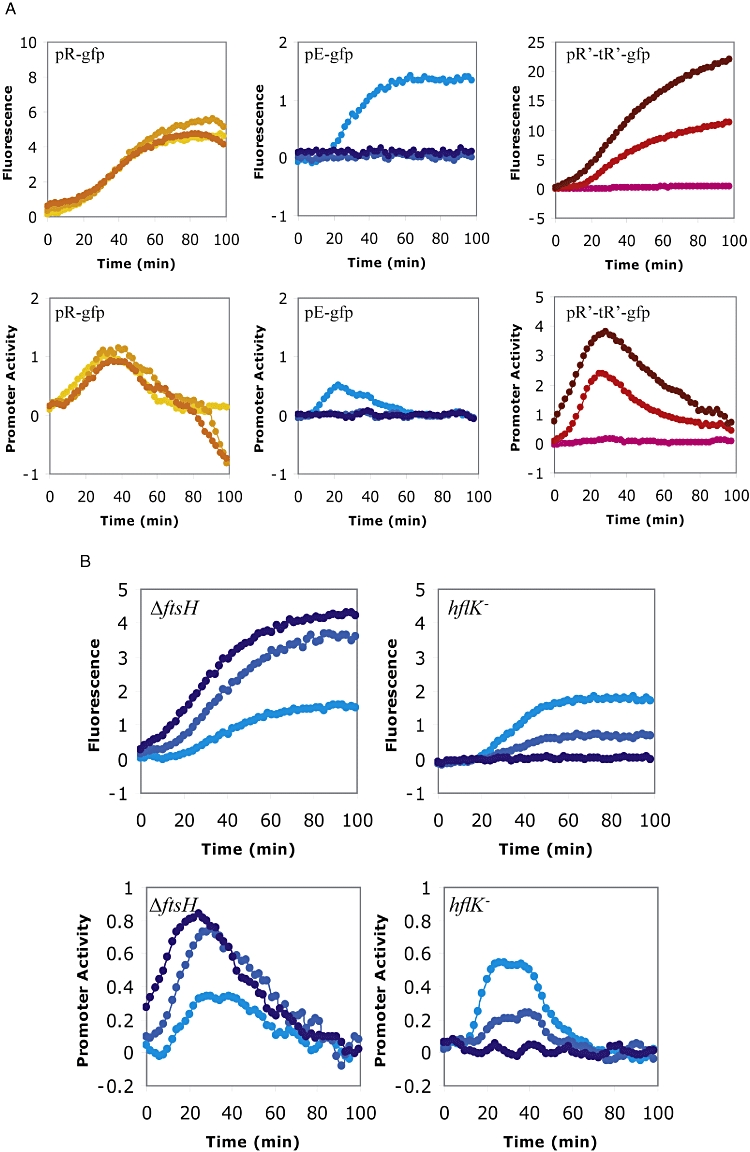
Prophage induction by heat shock at 42°C. A. Strains W3110 (λ*cI*_*857*_*kn*^*R*^) carrying the pR-gfp (orange), the pE-*gfp* (blue) or the pR′-tR′-*gfp* (red) plasmids were grown exponentially in minimal medium, heat induced at 42°C for 5, 10 or 15 min (light to darker colours respectively), and assayed for fluorescence at 32°C for the indicated times. The onset time of the graphs describing fluorescence levels (top) and promoter activity (bottom) is the transfer to 32°C. B. Strains A8926 (λ*cI*_*857*_*kn*^*R*^) deleted for the *ftsH* gene (left) and A9855 (λ*cI*_*857*_*kn*^*R*^) deleted for the *hflK* gene (right) carrying the pE-*gfp* plasmid were grown exponentially in minimal medium, heat induced at 42°C for 5, 10 or 15 min (light to darker colours respectively), and transferred to 32°C (time 0 min). Fluorescence activity (top) and promoter activity (bottom) are shown. The activity of pE–GFP was monitored following heat induction.

**Fig. 3:**
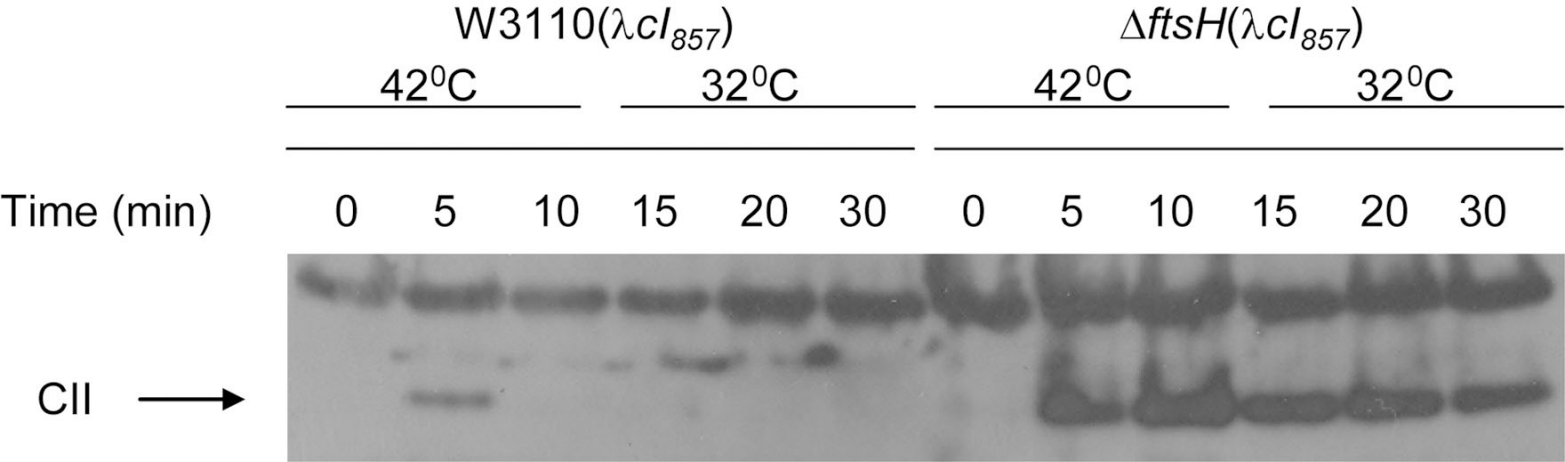
CII levels at heat induction. Strains W3110 (λ*cI*_*857*_*kn*^*R*^) and A8926 (λ*cI*_*857*_*kn*^*R*^) were grown overnight in Luria–Bertani at 32°C and then diluted 1:100 into 10 ml minimal media and grown to OD_600_ of 0.3. The cultures were transferred to 42°C for 10 min and were then returned to 32°C. Samples were taken at the indicated times from the onset of heat. Samples were loaded on 4–12% NuPAGE (Invitrogen), and CII levels were detected by Western blot using antibodies raised against the full-length CII (Kobiler *et al*., 2002).
- Expression from pR′-tR′–GFP shows that Q activity appears and increases following a heat treatment of at least 10 min; a 5 min heat pulse did not induce Q activity. The observed absence of Q activity after the first 5 min heat treatment agrees with two previous reports: First, commitment to phage DNA replication and late genes expression require more than 5 min heat induction (Weisberg and Gallant, 1967). Second, for anti-termination of transcription from pR′, a threshold level of Q gene product must be built up (Thomas *et al*., 1967).

To probe the induction process, we studied cells lysogenic for λ carrying the *cI*_*857*_ allele coding for a temperature-sensitive CI repressor (Sussman and Jacob, 1962). The transfer of these cells from 32°C to 42°C leads to rapid and coherent induction and expression of prophage genes. We introduced into these cells reporter plasmids containing either pR–GFP to monitor the inactivation of the CI repressor, pE–GFP to follow CII activity or pR′-tR′–GFP to monitor the anti-termination activity of Q. We have previously shown that expression from the pE–GFP and pR′-tR′–GFP reporters is absolutely dependent on the presence of CII and Q respectively (Kobiler *et al*., 2005). Lysogenic cells were transferred for 5, 10 or 15 min to 42°C, and then returned to 32°C for the monitoring of GFP levels. In a control experiment, heat treatment of λ*cI*^+^ lysogenic cells carrying the wild-type repressor showed no effect on GFP activity from any of the three promoters (data not shown). The results, shown in Fig. 2A, can be summarized as follows:

- Heat treatment leads to derepression of the pR–GFP fusion, resulting in a rapid rise of GFP fluorescence (Fig. 2A). The level of expression, measured after lowering the temperature, was not influenced by the duration of heat induction. Presumably, a 5 min heat pulse is sufficient for full inactivation of the CI repressor and allows transcription from pR present on the reporter plasmid. Note that the rate of expression from the pR promoter is reduced at around 40 min, presumably as a result of the occupation of the cellular transcriptional and translational machinery by the phage lytic network (Kourilsky and Knapp, 1974).
- Heat treatment of the lysogen for 5 min leads to CII activity as measured by the pE–GFP fusion (Fig. 2A). As detected by Western blot, the CII protein made during the first 5 min at 42°C is eliminated during the next 5 min at 42°C (Fig. 3). This elimination of CII protein explains the lack of CII activity after the 10 or 15 min heat pulse.

### FtsH and HflKC are responsible for the elimination of CII following heat induction but not following SOS induction

The *cII* and *Q* genes are co-transcribed from the pR promoter and *cII* is upstream of *Q* in the operon. During induction of λ*cI*_*857*_ lysogens, Q activity builds up while CII does not, suggesting a specific post-transcriptional inactivation of CII (Fig. 2A). FtsH is a heat-shock protease (Herman *et al*., 1995; Tomoyasu *et al*., 1995), which is known to degrade CII (Herman *et al*., 1993; Kihara *et al*., 1997; Shotland *et al*., 1997). We tested whether FtsH is responsible for eliminating CII following heat induction. We assayed CII activity in an *ftsH*-deleted lysogenic host following heat treatment, and found that CII activity was increased and extended relative to the *ftsH*^+^ host. The increase correlates with the duration of the heat treatment (Fig. 2B).

FtsH activity is known to be regulated by HflKC (Kihara *et al*., 1997), and mutations in the *hflKC* genes are known to favour the lysogenic response (Belfort and Wulff, 1973a) by increasing the half-life of CII (Belfort and Wulff, 1973b; Banuett and Herskowitz, 1987; Kihara *et al*., 1997). In agreement with the *ftsH* mutant results, we found a high CII activity following heat induction of λ*cI*_*857*_ prophage in an *hflK* mutant host (Fig. 2B).

To test whether the HflKC and FtsH functions are essential for efficient phage production following heat induction, λ*cI*_*857*_ lysogenic cells were grown and induced for 10 min at 42°C under identical conditions to those described above, and phage yield was assayed following 100 min at 32°C. The results show that the yield of mature phages was about 10-fold lower in the *hflK* mutant (1.8 phage/cell compared with 17 phages/cell for the wild-type parent host). If this behaviour were due to the accumulation of CII, one would expect that a prophage mutated in the *cII* gene would be insensitive to the absence of these host functions. Indeed, following induction of lysogenic cells carrying λ*cI*_*857*_*cII*_*41*_ (Belfort *et al*., 1975), the presence or absence of HlfKC function no longer affected the phage yield appreciably (20 phages/cell in the mutant compared with 27 phages/cell for the wild-type host).

### Prophage gene expression following SOS induction

**Fig. 4:**
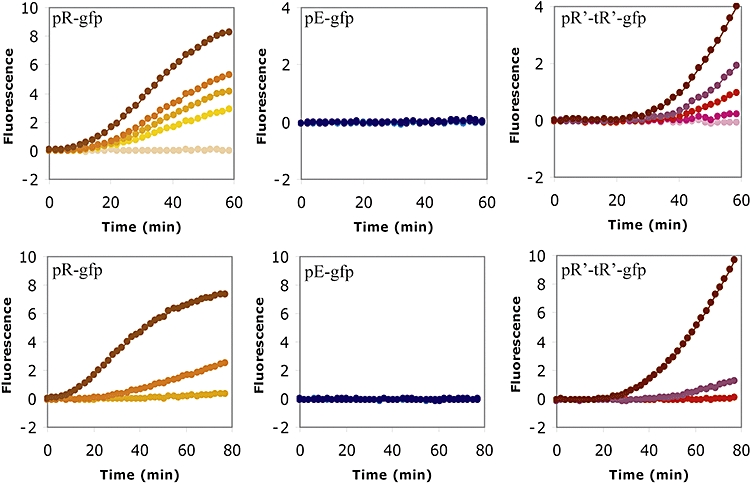
Induction of lysogenic cells by UV irradiation or by mitomycin C. Strains W3110 (λ*c*^+^*kn*^*R*^) carrying the pR-gfp (orange), the pE-*gfp* (blue) or the pR′-tR′-*gfp* (red) plasmids were irradiated with increasing UV doses (1, 5, 7.5, 10 and 20 J m^−2^), and GFP expression was followed at 32°C (top). Identical experiments were carried out following addition of mitomycin C at the concentration of 0.1, 1 and 10 mg ml^−1^, and fluorescence levels were then followed at 37°C (bottom). Light to darker colours represent increasing inducing doses.

Lysogenic cells can be induced by DNA-damaging treatments such as UV irradiation or mitomycin C; both treatments lead to activation of the SOS response (Sassanfar and Roberts, 1990). In order to investigate the phage response following SOS induction, lysogenic cells carrying the wild-type prophage were induced with increasing doses of UV or mitomycin C (Fig. 4). The results show that higher levels of GFP expression from the pR and the pR′-tR′ reporters are obtained with increasing UV doses or higher mitomycin C concentrations. We have recently shown in single cell analysis that pR at low UV levels is activated in the majority of the induced cells (Amir *et al*., 2007). On the other hand, these conditions lead to activation of the pR′-tR′ only in a fraction of the cells, in which pR activity is significantly lower. Interestingly, no expression from the pE promoter was observed at any of the inducing doses (Fig. 4). Similarly, no CII protein was detected by Western blot analysis (not shown). This is in contrast to heat induction, where CII activity was detected after short (5 min) heat treatment (Figs 2B and 3).

**Fig. 5:**
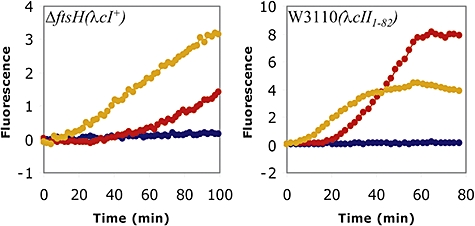
Elimination of CII by UV irradiation is unaffected by mutations in OOP or FtsH. The strains W3110 (λ*cII*_*1-82*_) and FtsH(λ*cI* ^+^) carrying the pR-gfp (orange), the pE-*gfp* (blue) or the pR′-tR′-*gfp* (red) plasmids were irradiated with 20 J m^−2^ UV and fluorescence levels were then followed at 37°C.

We tested if the activity of FtsH also underlies the absence of CII during SOS induction by assaying CII activity following UV irradiation or addition of mitomycin C to the *ftsH*-deleted host lysogenic for λ*cI*^+^. In these experiments, CII-mediated pE–GFP expression was not restored (Fig. 5). Furthermore, deletion mutants of several other protease-coding genes tested (*lon*,*htpX*, *clpY* or *clpQ*) did not restore CII activity either.

### The effect of the Cro repressor on CII activity following prophage induction

**Fig. 6:**
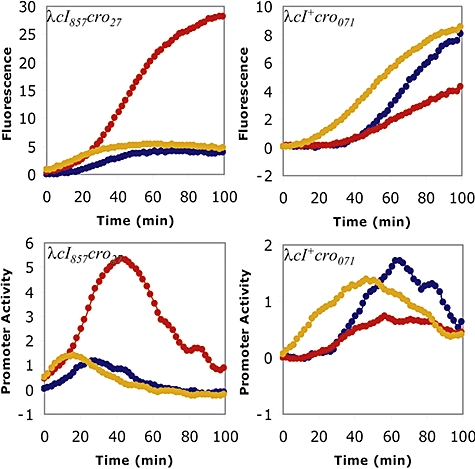
A mutation in the *cro* gene increases CII activity following heat-shock induction or SOS induction. Cells lysogenic for λ*cI*_*857*_*cro*_*27*_ were induced for 10 min under the experimental conditions described and coloured as in Fig. 2 (left). 10 mg ml^−1^ mitomycin C were added to cells lysogenic for λ*cI*^+^*cro*_*071*_ and fluorescence levels were then followed at 32°C (right). Fluorescence levels (top) and promoter activity (bottom) are shown.

The Cro function reduces transcription from both pR and pL promoters (Fig. 1B). Induction of a prophage carrying a mutation in the *cro* gene is predicted to lead to higher, continuous transcription of both the *cII* and the *cIII* genes. As expected, significant CII function was observed following either heat or mitomycin C induction of a *cro*^-^ lysogen (Fig. 6). Following mitomycin C induction, the appearance of CII activity is delayed and coincides with Q activity. The results suggest that the absence of CII in *cro*^+^ prophages either following prolonged heat treatment or SOS induction could be because there is only a limiting amount of CII made under these stress-induced conditions, and it is rapidly degraded. The increase in CII expression in a *cro*^-^ mutant overcomes its downregulation and allows CII activity to be measured.

### The absence of CII activity after SOS induction cannot be attributed to the CII antisense OOP RNA

The OOP antisense RNA stimulates the degradation of the CII mRNA by RNase III (Krinke and Wulff, 1990; Krinke *et al*., 1991). We therefore tested if mutations that affect the OOP RNA can overcome CII inhibition following SOS induction. Neither a mutant prophage defective in the *pOOP* promoter (Krinke and Wulff, 1990) nor a partial deletion mutant of the *oop* RNA gene (λ*cII*_*1-82*_) (Kobiler *et al*., 2002) restored CII activity to any detectable level after UV induction (Fig. 5). Moreover, an *rnc*^-^ mutant, defective in RNase III, failed to show CII activity following SOS induction (data not shown). These observations suggest that the OOP RNA has a minor role, if any, in the elimination of CII following SOS prophage induction.

## Discussion

Lambdoid bacteriophages utilize the same genetic network during infection and during prophage induction. However, following efficient prophage induction, lysis strongly predominates over the lysogenic pathway. This behaviour occurs even during growth in nutrient-rich media where bacteria contain more than one prophage copy of the λ genome. During infection by two or more λ phages, sufficient CII activity is produced to stimulate the lysogenic response. Nevertheless, an induced cell containing two or more λ prophages follows a complete lytic response. Our results suggest that for efficient lytic development of an induced lysogen, CII activity is downregulated following induction. Thus, the inactivation of CII as well as CI may be required to ensure a complete lytic outcome after induction (Oppenheim *et al*., 2005). Even if CII were available following induction, its presence could not maintain a lysogen in the absence of CI. In fact, CII may likely cause abortive lytic growth by blocking the Q-dependent switch to late lytic transcription; CII could not restore repressor levels because of continuous destruction of CI after SOS induction.

Although both heat and SOS induction lead to the same lytic course, our results show qualitative and quantitative differences in the activity of the developmental regulators.

While the pR promoter is immediately and fully activated even after a short heat induction, which completely inactivates the heat-sensitive CI protein, SOS induction shows a long lag and a dose-dependent expression from pR. A similar dependence on UV dosage was observed in individual cells (Amir *et al*., 2007). The longer lag in SOS induction, in contrast to heat induction, may be caused by the time required to generate the inducing signal after DNA damage (Sassanfar and Roberts, 1991).

CII activity was observed to be dependent on the duration of heat induction, with only the shortest 5 min exposure showing any CII activity. This led to the hypothesis that a short, 5 min incubation at the elevated temperature would lead to CI repressor inactivation, without an efficient induction of the heat-shock response, a condition that would allow some accumulation of CII. Longer incubations at 42°C fully induce the heat-shock response, leading to an increase in FtsH that rapidly degrades CII as it is being synthesized. In contrast, no CII activity was observed after any level of SOS induction. However, when CII is overproduced in a *cro*^-^ mutant prophage, CII activity is present both under SOS and prolonged heat induction, suggesting that inactivation of CII by each of these induction processes is a post-transcriptional event. It is clear that FtsH protease eliminates CII after heat treatment. However, neither FtsH nor several other protease functions tested affected CII activity following SOS induction. If proteases are responsible for the lack of CII activity following SOS induction, they remain to be identified.

Intriguingly, while both long heat treatment and DNA damage lead to a similar final outcome, different molecular mechanisms appear to be responsible for the processes. Results obtained by heat and SOS induction should not be treated equally without careful consideration of these differences.

We found that the lambda induction process is more complicated than a simple ‘on’ and ‘off’ switch of the CI repressor activity. The induction process takes place under conditions in which a number of phage and bacterial networks interact. Our findings clearly demonstrate that there are interactions between the lambda and host stress networks beyond the initial induction event. The absence of CII function, particularly in the SOS network, deserves further investigation to identify the participating elements of the networks and their role in the prophage induction process. Understanding of these processes should help improve theoretical models of lambda systems biology.

## Experimental procedures

### Bacterial strains, phages and plasmids

All bacterial strains are derivatives of W3110. The FtsH deletion strain used (A8926) is W3110Δ*ftsH3::kn*^*R*^*sfhC21 zad220::Tn10* (Tatsuta *et al*., 1998). The *hflK* mutant strain is W3110 *hflK*::*Kn*^*R*^ prepared by P1 transduction (Banuett *et al*., 1986). The phage strains are from our collection (Oppenheim *et al*., 2004; Kobiler *et al*., 2005). The reporter plasmids used in this work were previously described (Kobiler *et al*., 2005), except for the pR plasmid, constructed by introducing the 130 bp pR promoter region defined by co-ordinates 37905–38034 on the λ sequence and containing oR1 and oR2 repressor binding sites, into the GFP vector used previously (Kobiler *et al*., 2005).

### Fluorescence assays

For induction experiments, the cells carrying the reporter *gfp* fusion constructs were grown overnight in Luria–Bertani medium with 100 mg ml^−1^ ampicillin at 32°C. Overnight cultures were then diluted 1:100 into minimal media (M9 supplemented with 0.5% glycerol, 100 mg ml^−1^ ampicillin and 0.1% casamino acids), and grown to OD_600_ of 0.3 at the same temperature. For heat induction, flasks were transferred rapidly to 42°C with shaking. Samples were taken and kept on ice and then dispensed as duplicates into a 96-well plate on ice. For UV induction, cultures were concentrated 10-fold and irradiated under a pre-calibrated UV lamp. Cultures were then diluted 1:10 as duplicates into a 96 well plate on ice. For mitomycin C induction, a final concentration of 10 mg ml^−1^ was added to the 96 well plate containing duplicates of cultures at 0.3 OD_600_. Fluorescence and optical density measurements were carried out as described (Kobiler *et al*., 2005), with the exception that the assays were carried out at 32°C.

For a given promoter (pR, pE or pR′-tR′), the average of the total output fluorescence values from duplicate wells was determined for each time point. Then, the average value obtained at each time point from un-induced cells (also in duplicate wells) was subtracted to cancel out the intrinsic background fluorescence from the bacterial cells. The promoter activity (the first derivative of the fluorescence values) was calculated as previously described (Kobiler *et al*., 2005). Because of the different methods of induction used, in all the figures, the zero time point was defined as the beginning of increase in pR activity of the corresponding experiment.
